# Using One Health assessments to leverage endemic disease frameworks for emerging zoonotic disease threats in Libya

**DOI:** 10.1371/journal.pgph.0002005

**Published:** 2023-07-26

**Authors:** Lauren N. Miller, Hatem Elmselati, Alanna S. Fogarty, Milad E. Farhat, Claire J. Standley, Hanan M. Abuabaid, Abdulaziz Zorgani, Omar Elahmer, Erin M. Sorrell

**Affiliations:** 1 Center for Global Health Science and Security, Georgetown University Medical Center, Washington, DC, United States of America; 2 National Centre for Animal Health, Tripoli, Libya; 3 Heidelberg Institute of Global Health, University of Heidelberg, Heidelberg, Germany; 4 National Centre for Disease Control, Tripoli, Libya; 5 Department of Microbiology and Immunology, Georgetown University Medical Center, Washington, DC, United States of America; Johns Hopkins Center for Health Security: Johns Hopkins University Center for Health Security, UNITED STATES

## Abstract

Continued emergence, re-emergence and spread of zoonotic diseases demonstrates the imperative need for multisectoral communication and joint coordination of disease detection and response. While there are existing international frameworks underpinning One Health capacity building for pandemic prevention and response, often guidance does not account for challenges faced by countries undergoing long-term conflict and sociopolitical instability. The purpose of this research was to identify Libya’s laboratory and surveillance networks and routes of inter- and multisectoral communication and coordination for priority zoonotic diseases. The One Health Systems Assessment for Priority Zoonoses (OH-SAPZ) tool is an established methodology that was adapted and applied to the Libyan context to support prioritization of zoonotic diseases, development of systems map schematics outlining networks of communication and coordination, and analysis of operations for targeted capacity building efforts. Five zoonotic diseases were selected to undergo assessment: highly pathogenic avian influenza, brucellosis, Rift Valley fever, leishmaniasis and rabies. Through decisive acknowledgement of Libya’s unique health setting, we mapped how patient and sample information is both communicated within and between the human, animal and environmental health sectors, spanning from local index case identification to international notification. Through our assessment we found strong communication within the public and animal health sectors, as well as existing multisectoral coordination on zoonotic disease response. However, local-level communication between the sectors is currently lacking. Due to the ongoing conflict, resources (financial and human) and access have been severely impacted, resulting in limited laboratory diagnostic capacity and discontinued disease prevention and control measures. We sought to identify opportunities to leverage existing operations for endemic diseases like brucellosis for emerging zoonotic threats, such as Rift Valley fever. Analysis of these operations and capabilities supports the development of targeted recommendations that address gaps and may be used as an implementation guide for future One Health capacity building efforts.

## Introduction

The number of emerging infectious disease (EID) outbreaks, epidemics, and pandemics has continued to increase over the last 70 years [1] with a majority attributed to zoonotic spillovers [2]. Factors such as global travel, trade, environmental and climactic conditions, large population densities, intensive agriculture, and the overuse of antibiotics have all served to accelerate disease emergence and spread [3]. While the majority of zoonotic EIDs are viral in nature, zoonotic spillover can also be caused by bacteria, parasites, or fungi [4]. In response to sustained recurrence of zoonotic threats, modern global health initiatives have adopted the One Health approach to health systems strengthening efforts and EID detection and response activities, purposefully integrating human, animal and environmental health sectors [5–7]. The Quadpartite Organizations, which includes the Food and Agriculture Organization of the United Nations (FAO), the United Nations Environment Programme (UNEP), the World Organisation for Animal Health (WOAH), and the World Health Organization (WHO), define One Health as an “integrated, unifying approach that aims to sustainably balance and optimize the health of humans, animals, plants and ecosystems. The approach recognizes that these are closely linked and interdependent, and mobilizes multiple sectors, disciplines and communities at varying levels of society to work together to foster well-being and tackle threats to health and ecosystems” [8].

Zoonotic disease threats are complex, as they are often multifactorial, can encompass a variety of species as reservoir and/or intermediate hosts, and may result in transboundary spread [9]. Therefore, robust and progressive One Health mitigation and response strategies require concerted efforts that first identify related networks and outline operational interdependencies between public health and all other relevant sectors [3]. Moreover, disruptions stemming from outbreaks and public health events often have severe ramifications to agricultural, environmental, trade, tourism, energy, civil protection, and/or transportation sectors in addition to healthcare systems. There is an evident need for holistic One Health approaches that capture the multidimensionality of national outbreak preparedness and response strategies, as well as adaptable frameworks and metrics designed to accommodate dynamic environments with differing capabilities. Our team has previously developed and deployed a methodology for assisting countries in assessing existing systems for One Health, with an emphasis on priority zoonotic diseases, and identifying opportunities for capacity strengthening or addressing gaps. The One Health Assessment for Priority Zoonoses (OH-SAPZ) tool has been applied in part or in full in Jordan, Egypt, Algeria, Guinea and Iraq and is included in the Surveillance and Information Sharing Operational Tool (SIS OT) developed by FAO, WHO, and WOAH [10, 11]. The OH-SAPZ methodology is a phased approach to engage human, veterinary and environmental health sectors in the development of a consensus priority zoonotic diseases list; uses case study scenario discussions to examine the structures and mechanisms for communication and coordination between and within governmental sectors for the creation of systems map schematics; and provides a framework for analyzing strengths and weaknesses of existing intersectoral coordination in order to help identify gaps and develop targeted recommendations to strengthen One Health capacity and coordination [12].

Prolonged and recurrent instability diminishes a country’s capacity to adequately care for its citizens both in short and long-term tenures through inadequate access to healthcare services and reduction to preventative public health measures [13]. The nation of Libya has faced ongoing armed conflict and perennial sociopolitical instability for over a decade, severely challenging health service provisions, resulting in limited or redirected funding, insufficiencies in the trained health workforce, and ultimately impacting the country’s ability to adequately prevent, detect and respond to infectious diseases. While a unified government was re-established in March 2021 [14], the prolonged governmental division resulted in legislative stalemates and a lack of whole-of-country approaches to implementing health system strengthening initiatives, particularly at the ministerial level. Thus, the emergence of severe acute respiratory syndrome coronavirus 2 (SARS-CoV-2) and the persistent spread of coronavirus disease 2019 (COVID-19) proved to exacerbate existing systematic weaknesses and further undermine health security capacities [15]. Libya’s geographic location also puts it at a high risk for emergence and re-emergence of zoonotic diseases as the country share borders with Egypt, Sudan, Niger, Chad, Tunisia and Algeria, increasing the opportunity for transboundary spread from movement of both humans and animals. The country grapples with illegal migration and illegal trade of animals from sub-Saharan African countries and Asian countries [16]. These risk factors introduce diseases vectors and potentially harmful pathogens into the country and consequently zoonotic diseases emergence. Libya is not unique in the multidimensional challenges stemming from long-term armed conflict and a politically-unstable landscape; nevertheless, there is an evident gap in health system strengthening frameworks that account for the complicated nature faced in both low-resource and socio-politically uncertain settings. The objective of our research was to apply the OH-SAPZ methodology in Libya, working collaboratively across sectors to prioritize zoonoses and assess current strengths and gaps, and identify opportunities for strengthening One Health systems related to these priority diseases.

## Methods

### Applying the OH-SAPZ to Libya

Using the OH-SAPZ tool described above, we adapted the prescribed, phased methodology to ascertain the existing surveillance and diagnostic networks in place, as well as systems for communication and coordination between key stakeholders in Libya. To accommodate the governance challenges at the national level and ever-changing political environment in Libya, we engaged with subnational-level stakeholders at the National Centre for Disease Control (NCDC), the National Centre for Animal Health (NCAH), and the Environment General Authority (EGA) who are directly responsible for leading and managing disease outbreaks across the country. The NCDC and NCAH’s longstanding Memorandum of Understanding (MOU) allowed for us to leverage cross-sectoral relationships and acquire valuable insight into the interdependent operations in place for zoonotic diseases in Libya. Due to the uncertainties caused by COVID-19, we modified our in-person assessment approach and shifted to remote correspondence and virtual videoconferencing engagements. Through the commitment from in-country partners, pre-existing partnerships, and the adaptiveness of the OH-SAPZ tool, we successfully completed the One Health assessment using these modified techniques.

### Stakeholder mapping

The first step was identification of and engagement with key stakeholders. Through consultation and previous collaborations in Libya, we identified three subnational government sectors, NCDC, NCAH and EGA, critical to our One Health assessment process. Next, we performed an in-depth search and literature review of zoonotic diseases present in Libya for consideration in the OH-SAPZ methodology. We first reviewed Programing for Monitoring Emerging Diseases (ProMed) online surveillance reports and outbreak notifications to WOAH’s World Animal Health Information System (WAHIS) from health authorities to determine the range of zoonotic diseases reported in Libya. Our search strategy included using ProMed’s “Search Post” function, and the application of programmed country filters in WAHIS Event Management database. Next, a literature review was conducted to summarize knowledge about the identified diseases and key capacities necessary for outbreak detection and response. In this literature review, we collected information pertaining to available vaccines, gold standard diagnostics and rapid diagnostic tests, as well as national and/or regional strategies for prevention and response, and relevant national and international stakeholders. A total of 53 resources, including peer-reviewed publications, international and public health agency factsheets, were referenced in the initial literature review. Building on our internal literature review findings, we further developed a comprehensive list of nine disease candidates (Table 1).

**Table 1:** Priority disease list and qualifying criteria used for selection. Application of qualifying criteria used for selecting priority zoonoses in Libya. Diseases noted with an asterisk were confirmed as priority diseases to undergo One Health assessment.

Qualifying Criteria	Brucellosis*	Leishmaniasis*	Rift Valley fever (RVF)*	Highly Pathogenic Avian Influenza (HPAI)*	Rabies*	Crimean-Congo hemorrhagic fever (CCHF)	Bovine tuberculosis	West Nile virus	Toxoplasmosis
**Endemic in country**	X	X			X		X		X
**Outbreak potential in country**			X	X		X	X	X	X
**Emerging in country**			X	X		X		X	
**Potential for endemic or pandemic in humans or animals**	X	X	X	X		X	X		X
**Pathogen for international concern–reportable to WHO**			X	X		X			
**Pathogen for international concern–reportable to WOAH**	X	X	X	X	X	X		X	
**Large disease burden in humans**	X			X		X			X
**Large disease burden in livestock or domestic animals**	X		X	X	X		X		X
**Large disease burden in wildlife**				X	X		X		
**Listed on MOH notifiable disease list**		X		X	X	X		X	
**Listed on MOA notifiable disease list**									
**Regional priority disease**	X	X		X	X				
**Available control strategies/programs**	X	X		X	X				
**Available laboratory diagnostics (central and sub-national level)**	X	X	X	X	X		X		X
**Mechanisms for improved stakeholder communication and coordination**			X	X	X	X		X	
**Available treatments**	X	X	X	X	X	X			X
**Economic or social impact**	X	X	X	X			X		X
**Bioterrorism potential**	X		X			X			

### Disease selection and prioritization

Through facilitated videoconference discussions, our team next presented a series of qualifying criteria to help narrow down the extensive zoonoses list and reach consensus on the five diseases to undergo the One Health assessment (Table 1). These qualifying criteria included endemicity, outbreak or pandemic potential, the burden of disease on humans and animals, social and economic impacts, available laboratory diagnostic capacities, as well as the causative pathogen’s bioterrorism potential [12]. In order to provide a tailored approach to prioritization and allow for adaptation at the national and subnational level, our methodology intentionally does not assign weights to the qualifying criteria. In this way, the final selection of a disease is not linked to a pre-determined priority that may not represent the national (or subnational) context. Through the application of these qualifying criteria, we provided a standardized method for narrowing down the disease priorities across our multisectoral focal points, which ultimately guided their final selection of five diseases to undergo One Health assessment.

### Case study-based scenario discussions

Following consensus agreement on the five priority zoonotic diseases, we developed case study scenarios for each disease and held virtual facilitated discussions with stakeholders. The goal of the scenario discussions was to acquire information on surveillance and laboratory capacities in Libya, as well as existing multisectoral coordination response mechanisms for each of the priority diseases; this included case identification and reporting mechanisms, specimen sample collection and submission, laboratory diagnostic and confirmation testing capabilities, case investigation and management protocols, and control measures for each sector. We constructed each case study by adapting historical descriptions of outbreaks and situations relevant to the Libyan context and designed them in a way to involve all three implicated sectors in each scenario discussion. While the scenarios were fictional, we incorporated steps in outbreak detection, assessment and response outlined in the OH-SAPZ tool [12]. Additional activities associated with disease management, such as social mobilization, risk communication, and advocacy, were added into relevant disease scenarios. We arranged two virtual meetings to review these case studies, including a scenario for each priority disease, and facilitated group discussions to collect the necessary qualitative data. Stakeholders from departments within NCAH, NCDC, and EGA, as well as representatives from relevant non-governmental organizations participated.

### Systems mapping

Using the information gathered from the case study scenario discussions, we developed five unique systems map schematics depicting the human, animal and environmental health sectors’ detection and response operations across different levels of governance. An editable PowerPoint template, standardized for the OH-SAPZ method, was used to create the map schematics. In each systems map schematic, we depicted the movement of patients, specimens and data, as well as the routes of communication and coordination within and between the sectors. We regularly consulted with scenario discussion participants throughout the map development process and obtained sector-wide approval of the final systems map schematics.

### Analysis and recommendations

After building the systems map schematics for the priority diseases, we reviewed each sector’s operations and determined the strengths and gaps in capacity, as well as any neglected opportunities for enhanced communication and coordination between NCDC, NCAH and EGA. The systems map schematics provided a direct visual of the existing structures and systems (as well as gaps) in place for coordination and communication both within and between the sectors. We also used peripheral data from case study scenario discussions to inform the analysis. The strengths (presence of capacity) and gaps (lack of capacity or lack of information) were categorized under: 1) detection; 2) laboratory capacity; 3) response; 4) prevention and control measures; and 5) communication and coordination. A cross-comparison between the five disease-specific systems map schematics also helped our team determine existing capacities and multisectoral coordination that could be leveraged and guided our team’s recommendation development. We worked with stakeholders to validate specific recommendations and action items to address gaps for each sector and each priority zoonotic disease.

## Results

### Priority zoonotic diseases in Libya

The disease prioritization process resulted in a list of five priority zoonotic diseases: brucellosis, leishmaniasis, Rift Valley fever (RVF), highly pathogenic avian influenza (HPAI) and rabies (Table 1). Consensus regarding the final list was reached without any major disagreements among our key stakeholders. All five diseases prioritized by our stakeholders are pathogens of international concern and reportable to WOAH; additionally, laboratory diagnostics and treatments are available in Libya for each of the selected diseases. Brucellosis is an endemic disease in both human and animal populations, such as cattle, camels, goats and sheep [17, 18].

Both cutaneous leishmaniasis (CL) and visceral leishmaniasis (VL) are also endemic, with nearly 1000 human CL cases reported each year since 1980, the majority resulting from zoonotic and anthroponotic transmission [19–21]. In comparison, RVF is an emerging zoonotic disease of concern, with the first animal outbreak reported by officials in December 2019 detected in a flock of sheep and goats [22, 23]. In 2014, Libya reported its first and only outbreak of HPAI H5N1 in poultry which spread to humans, resulting in 10 cases and 5 fatalities [24, 25]. Finally, as in other North African countries, rabies is endemic in Libya; however, there is limited information on prevalence in human and animal populations due to limited surveillance and underreporting [26, 27]. While the country has previously declared itself free of canine rabies, in February 2022 alone the NCDC reported 4 human cases [28].

**Fig 1:**
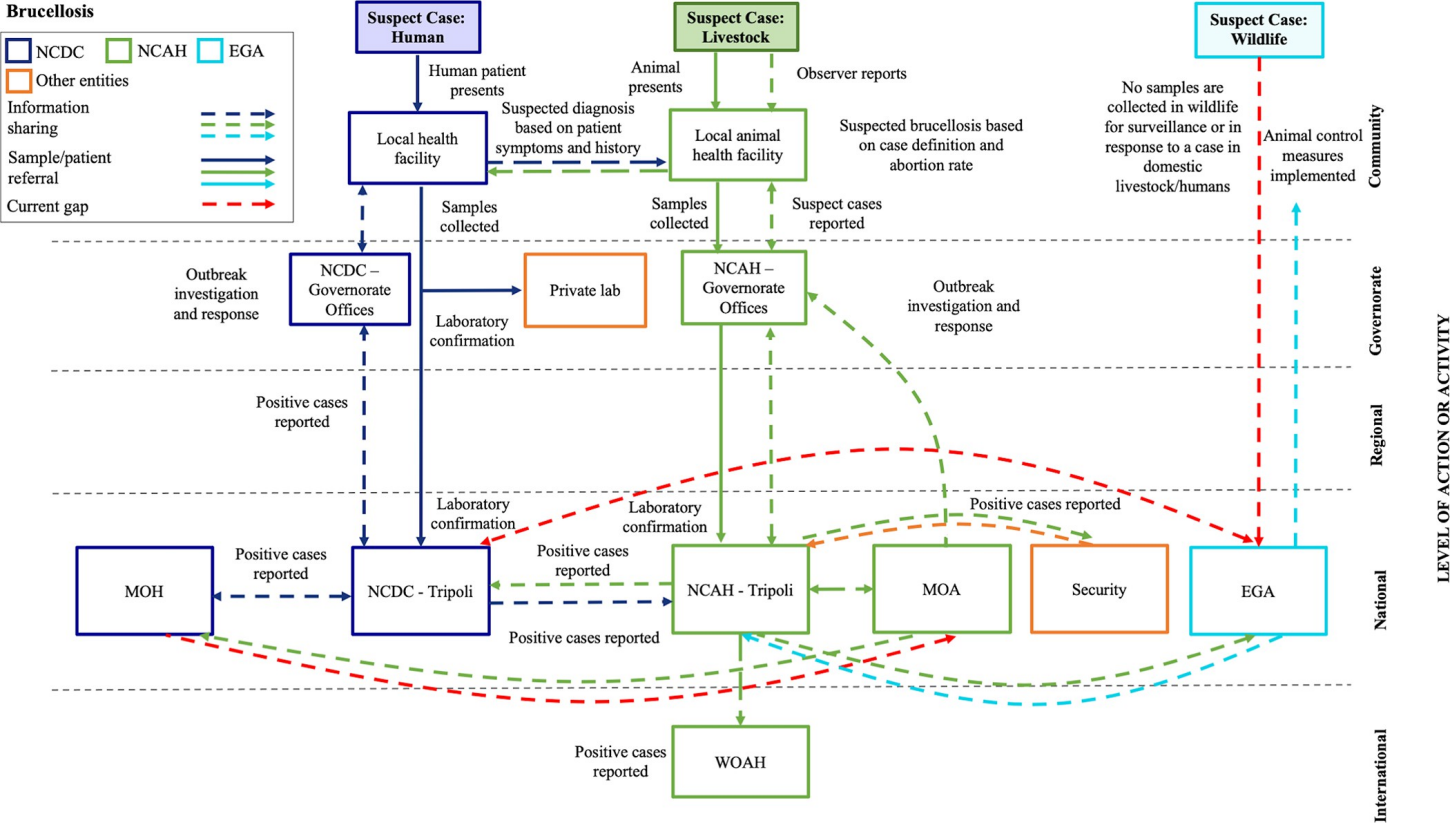
Systems Map Schematic for brucellosis from the community level (top) to the international level (bottom). The figure depicts a flow chart schematic of surveillance and laboratory mapping for brucellosis. Efforts in surveillance and response led by NCDC are represented in dark blue while those led by NCAH and EGA are in green and light blue, respectively. Abbreviations: NCDC = National Centre for Disease Control; NCAH = National Centre for Animal Health; EGA = Environment General Authority; MOH = Ministry of Health; MOA = Ministry of Agriculture; MOE = Ministry of Environment. Solid arrows represent sample/patient sharing. Arrows with dashes represent information sharing. Dark blue arrows indicate human cases, samples, and/or shared information whereas green and light blue arrows show animal-related information and wildlife-related information, respectively. Red arrows with dashes indicate current gaps.

**Fig 2:**
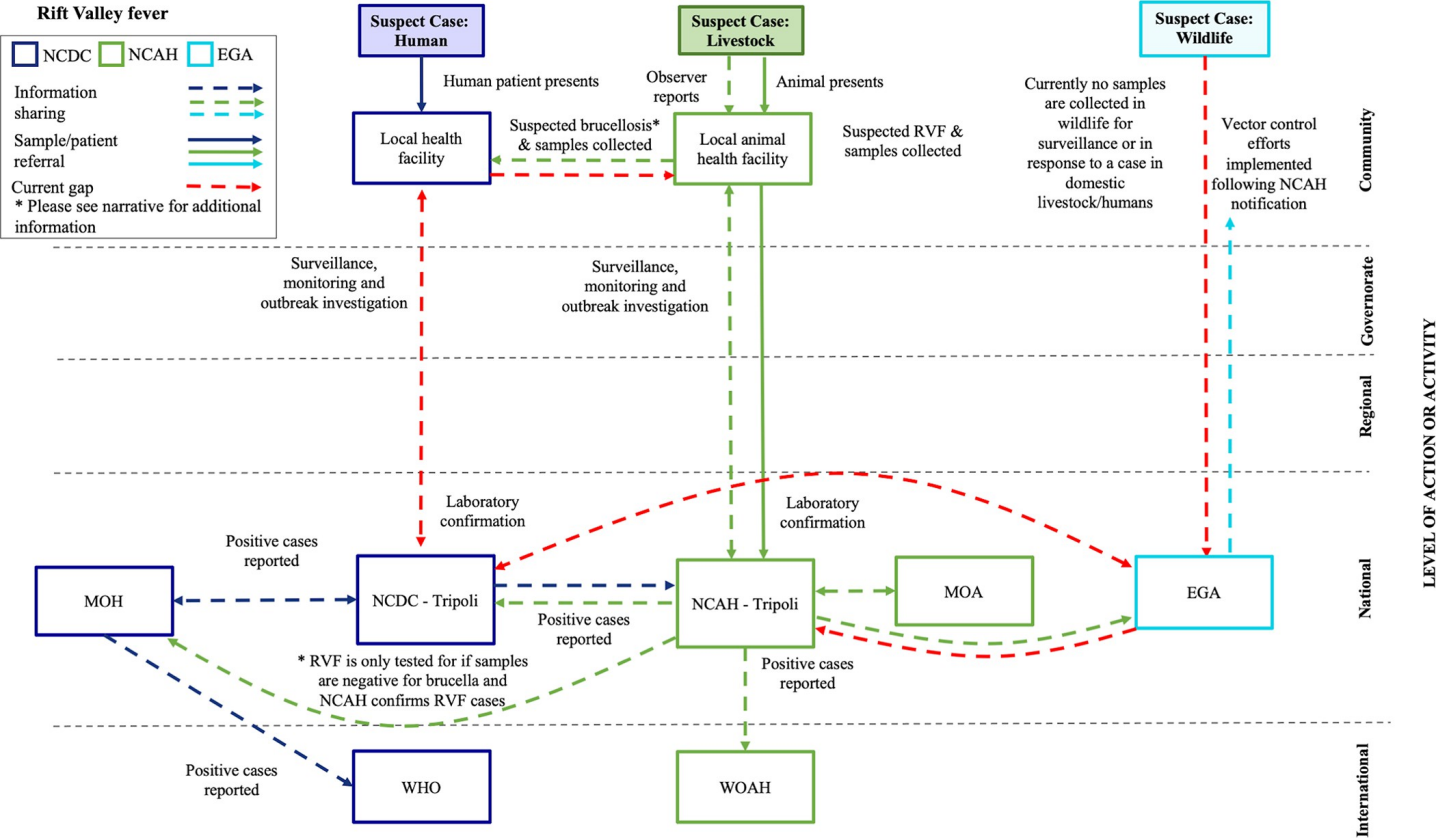
Systems Map Schematic for Rift Valley fever (RVF) from the community level (top) to the international level (bottom). The figure depicts a flow chart schematic of surveillance and laboratory mapping for RVF. Efforts in surveillance and response led by NCDC are represented in dark blue while those led by NCAH and EGA are in green and light blue, respectively. Abbreviations: NCDC = National Centre for Disease Control; NCAH = National Centre for Animal Health; EGA = Environment General Authority; MOH = Ministry of Health; MOA = Ministry of Agriculture; MOE = Ministry of Environment. Solid arrows represent sample/patient sharing. Arrows with dashes represent information sharing. Red arrows with dashes indicate current gaps.

Here, we present in detail the systems map schematics for brucellosis and RVF (Figs 1 and 2) and highlight how structures and systems in place for endemic diseases can be leveraged to support emerging zoonotic disease surveillance, detection and response capacities. With the rising concern of newly emerging zoonoses and the need for sensitive surveillance and rapid response processes, there may be opportunities to leverage existing monitoring systems for endemic diseases and experiential knowledge from seasoned experts across various sectors to help assess epidemiological patterns, trends and timely detection of changes, as well as determine the need for interventions. Systems map schematics for leishmaniasis, HPAI and rabies are available for reference in S1–S3 Figs. It should be noted that while EGA was a key stakeholder in our systems mapping process, their role in detection, surveillance and response to the priority diseases was minimal due limited human and financial resources that severely hinder involvement in wildlife surveillance and outbreak response activities.

#### Brucellosis and RVF, the Libyan context

Libya is at high risk for future brucellosis and RVF outbreaks, as it borders endemic and enzootic countries Niger, Sudan, and Egypt and livestock trade movement, both legal and illegal, is known to occur [23]. The majority of cases are attributed to intensive importation of livestock for breeding [17]; sheep and goats play an important role in religious and cultural festivals [29]. The last reported outbreak of brucellosis in 2020 was in a herd of 36 cattle, resulting in nine cases and one death [30]. Serological evidence from the first reported outbreak of RVF in 2019 indicates the virus was introduced from a neighboring country through livestock trade [22, 23, 31]. Previous studies, however, propose the virus was circulating in the country long before this outbreak was detected however no human cases or deaths have been reported to date [23].

### Systems assessment

#### Brucellosis key findings

We identified strong existing communication and coordination at the local levels within and across NCDC and NCAH and challenges in reporting cases up to subnational and national-level authorities. Both the human and animal health sectors demonstrated strengths in case detection and sample collection; however, we identified gaps in NCDC’s case reporting and both sectors’ surveillance capacities. At NCDC, cases of brucellosis are not reported directly to NCDC through the existing Early Warning Alert and Response Network (EWARN) system but through routine surveillance; reports are shared weekly with governorate offices. Officials relayed that case reports received by the Tripoli office are not always completed, validated or confirmed. When NCDC has reports of suspected cases they coordinate, informally, with counterparts at NCAH; however, following case confirmation, the NCDC Director shares reports and formally coordinates with NCAH on response. Both NCDC and NCAH demonstrated strong diagnostic and confirmation testing capacity at the national reference laboratories in Tripoli. However limited capacity for enzyme-linked immunosorbent assay (ELISA) and polymerase chain reaction (PCR) exists at the governorate level requiring clinical samples to be sent to the national reference laboratory in Tripoli or to private laboratories where test kits are more readily available. In most cases ELISA is most often used for confirmation, Rose Bengal tests (RBTs) are uncommon and PCR is not used for human diagnosis due to a lack of access to diagnostic test kits. At present, there is no formal agreement between NCDC and private laboratories to assist with diagnostic testing or to report results. While we identified a strong multisectoral approach to brucellosis response and disease control efforts, NCDC lacks an approved national strategy and a designated program responsible for implementing preventive measures, risk communications, and outbreak management. In response to an outbreak, NCDC deploys a multidisciplinary team from Tripoli to manage and lead the response activities. NCAH, in comparison, has well-established outbreak investigation and response management processes in place for brucellosis and is in the processes of establishing an official rapid response team (RRT) to lead local investigation and response. NCAH employs numerous countermeasures for brucellosis containment and control including, culling and disposing of infected animals, cleaning, disinfecting, restricting animal movement, discontinuing distribution or heat-treating milk products, and performing medical examinations on farm workers. Nevertheless, preventive and control measures are significantly strained by the costly procurement of vaccines and compensation to farmers for culling. While NCAH offers vaccines to farmers free of charge, access to supply and adequate distribution are major challenges. The national vaccine program successfully implemented in the 1980’s was discontinued when case thresholds were not met. According to NCAH policy, however, the threshold for implementing a brucellosis vaccination program is when cases are reported in more than 1% of animal population. Therefore, the brucellosis vaccine policy is not represented in practice. NCAH is currently working with FAO to develop national immunization strategies for brucellosis control, which have been referred to the Ministry of Agriculture (MOA) for approval and budgetary allocation. It was difficult to acquire information from EGA and confirm their lack of a formalized role in prevention, detection and surveillance, as well as response activities for brucellosis in Libya.

#### Brucellosis key recommendations

Our team consulted with key stakeholders to review the findings from our assessment of brucellosis and develop recommended action to address these gaps. Several key actions were identified to increase NCDC’s laboratory capacity, such as procurement and distribution of RBT and ELISA across Libyan governorates to support the rapid screening and detection of brucellosis. In addition, PCR test kits should be provided to select governorate-level laboratories, particularly in high-risk areas, to support the establishment of regional testing centers for rapid detection. There is also a need for NCDC to establish a formal agreement for coordination with private laboratories to bolster access to *Brucella* diagnostic testing methods and enhance the sharing of results. Finally, our recommendations reinforced the importance of multisectoral RRT coordination between NCDC and NCAH, as well as local-level capacity building, such as outbreak management training for community health officials to support on-the-ground case investigation and outbreak response efforts. A detailed list of the recommendations and action items developed for brucellosis can be found in Table 2.

**Table 2:** Brucellosis gap analysis key findings and recommendations. The table describes the key findings that were identified from the gap analysis assessment performed on the brucellosis systems map schematic and other relevant information collected during the case study scenario discussions. Findings are separated based on the implicated sector and organized into five categories related to detection, laboratory capacity, response, prevention and control measures, and communication and coordination. We developed recommended actions to address the identified gaps or lapses in capacity. Finally, we included recommended timelines for each target action, which include short, mid and long-term implementation to support action planning processes. While EGA’s operations were included in the systems map schematic, their limited scope and capacity prevented inclusion in this analysis.

Subnational Sector	Category	Key Findings	Recommended Action	Recommended Timeline for Implementation Short-term = <6 months; Mid-term = 6–12 months; Long-term = >12 months
NCDC	Detection	Cases of brucellosis are not reported from the local level directly to NCDC through the EWARN system nor through weekly zoonotic disease reports shared with governorate offices and NCAH.	Develop (if not available) and distribute guidelines for reporting suspect/positive brucellosis cases to local level clinicians, laboratories and health officials to increase case reporting and surveillance.	Short-term
	Laboratory Capacity	At NCDC, ELISA and RBTs are uncommon.	Acquire and/or expand access to ELISA and RBTs across Libyan governorates, starting with high-risk areas, for rapid detection and screening of brucellosis.	Mid-term
		Limited testing capacity at the governorate level for PCR.	Identify and map laboratories in high-risk governorates with adequate capacity for PCR.	Mid-term
			Acquire PCR test kits for high-risk governorate laboratories to expand access and establish regional brucellosis testing capacity.	Long-term
		No formal agreement between NCDC and private laboratories to assist with diagnostic testing or share results.	Establish formal coordination with private laboratories to bolster access to brucella diagnostic testing and reporting.	Mid-term
	Response	NCDC lacks an office or program dedicated to brucellosis, nor is there a specific strategy for outbreak response.	Establish a national brucellosis program at NCDC responsible for outbreak strategy, prevention/risk communication, detection, response, and control efforts.	Long-term
	Response	Multidisciplinary outbreak response team is activated and sent from Tripoli to the affected governorate(s).	Train local level health officials on brucellosis outbreak management and response measures to support case investigation and management.	Long-term
	Prevention/Control Measures	No known educational awareness campaigns	Implement educational awareness campaigns for the general public on risks for exposure	Short-term
NCAH	Response	Current RRT teams deployed from Tripoli to the governorates	Finalize and train the official RRT to lead local investigation and response	Mid-term
			Conduct trainings for local animal health officials, particularly in high-risk areas, on outbreak investigation to support response.	Long-term
	Prevention/Control Measures	No national immunization policies in place for brucellosis.	Formalize a national immunization policy for brucellosis control with MOA.	Mid-term
			Coordinate with MOA to allocate and acquire necessary funding to support brucellosis regular immunization campaigns. Re-establish vaccine access to livestock farmers.	Long-term

#### RVF key findings

Our team identified strong existing communication and coordination between NCDC and NCAH when a case of RVF is confirmed. A positive case is immediately reported by the Ministry of Health (MOH) to WHO as outlined in Annex 2 of the International Health Regulations (IHR) (2005) [5]. Regardless of strong multisectoral coordination, our team identified a number of significant gaps in NCDC and NCAH’s detection, prevention and control capacities for RVF. When a patient presents with clinical symptoms similar to influenza-like illness (ILI), clinical diagnosis favors brucellosis due to its endemicity. When diagnostic tests come back negative for *Brucella*, oftentimes patients are then diagnosed with pyrexia of unknown origin (PUO). The consideration of RVF as a viable diagnosis is based on clinical judgement, a negative *Brucella* test, and awareness of active animal cases. Neither NCAH or NCDC currently conduct active or passive surveillance for RVF. While NCDC recognizes the presence of *Aedes* and *Culex spp*. Mosquitoes in country and the risk for human exposure to vector-borne diseases, there is no official vector control program or routine spraying for mosquitoes; any vector control efforts led by EGA (desterilization, fogging and spraying in communities) are implemented in high-risk areas following reports from NCAH or initiated following complaints directly from the community. NCAH has developed policies and procedures to control the movement of infected animals, farmers and their equipment during an active RVF outbreak. As RVF was only recently detected in Libya, technicians from Tripoli deploy to assist local authorities with animal movement restriction and implementation of infection control mechanisms to reduce transmission. Compensation to farmers for culled, infected animals is mandated by law, however, is not currently being implemented due to budgetary restraints. Farms that do not have infected animals are recommended to undergo vaccination of susceptible animals; however, due to lack funding, vaccination does not occur. Finally, we learned that neither NCDC nor NCAH include RVF in disease prevention public awareness or educational campaigns. Officials shared that based on their experience, there is an overall lack of knowledge and awareness of transmission risks in high-risk groups and the general population.

#### RVF key recommendations

From the assessment performed on NCDC, NCAH and EGA’s networks and operations for RVF, we developed several key recommendations to strengthen multisectoral detection and surveillance, as well as prevention and control measures. As previously mentioned, patients are not being tested for RVF, particularly across local health facilities; we recommended that NCDC develop diagnostic guidance for healthcare workers when considering ILI clinical manifestations. To enhance One Health detection and adequate response to RVF, local health care facilities and veterinary facilities should communicate positive cases to support coordinated surveillance and outbreak response. Finally, in an effort to support increased public awareness, NCAH and NCDC should establish routine public awareness campaigns for vector-borne diseases, including RVF, targeting high-risk populations, such as farmers, butchers and veterinarians, that includes risk communication messaging, transmission risks factors, symptoms and prevention measures. A detailed list of the recommended action items that were developed for RVF can be found in Table 3.

**Table 3:** RVF gap analysis key findings and recommendations. The table describes the key findings that were identified from the gap analysis assessment performed on the RVF systems map schematic and other relevant information collected during the case study scenario discussions. Findings are separated based on the implicated sector and organized into five categories related to detection, laboratory capacity, response, prevention and control measures, and communication and coordination. We developed recommended actions to address the identified gaps or lapses in capacity. Finally, we included recommended timelines for each target action, which include short, mid and long-term implementation to support action planning processes. While EGA’s operations were included in the systems map schematics, their limited scope and capacity prevented inclusion in this analysis.

Subnational Sector	Category	Key Findings	Recommended Action	Recommended Timeline for Implementation ST = <6 months; MT = 6–12 months; LT = >12 months
NCDC	Detection	Clinical diagnosis of RVF is absent at most health facilities	Develop/update SOPs for healthcare professionals that includes RVF case definitions, safe sample collection, and guidance for testing patients who display clinical symptoms of fever and are negative for *Brucella*.	Short-term
			Develop educational materials/factsheets for healthcare workers that compare RVF and brucella infections and diagnosis considerations.	Short-term
	Prevention/Control Measures	No public awareness/educational campaigns	In coordination with NCAH, develop routine public awareness campaigns targeting high-risk populations, farmers, butchers and veterinarians, that includes risk communication messaging on RVF transmission risks, symptoms and prevention.	Short-term
	Prevention/Control Measures	Lack of active surveillance or official vector control program with EGA	Develop a multi-sectoral technical working group on vector control for all priority vector-borne diseases in Libya that is responsible for coordinating resources, surveillance, and spray campaigns.	Mid-term
	Communication and Coordination	Lack of local-level communication between public health and animal health facilities on confirmed cases	Established and formalized process for communication from local public health to animal health facilities in response to a confirmed case.	Short-term
NCAH	Detection	No known surveillance of RVF	Plan and conduct a joint seroprevalence study with NCDC to determine current exposure rates in animals in high-risk governorates.	Long-term
	Response	Lack of capacity to manage investigation/response/control measures at the local level	Develop and implement trainings for local animal health officials to support outbreak investigation and response that align with national guidelines.	Long-term
	Prevention/Control Measures	Farmers do not receive compensation for culled livestock	Allocate and approve necessary funds to compensate farmers during outbreak response.	Long-term

Both NCDC and NCAH demonstrated critical strengths in their operational capabilities and networks for detection and response to endemic and emerging disease threats. While we present the outcomes for two of the five priority diseases (see S1–S3 Figs for the remaining priority disease maps), we found commonalities in sector strengths and weaknesses across the endemic and EIDs (Table 4).

**Table 4:** NCDC and NCAH capacity strengths and weaknesses for endemic and emerging infectious diseases. Table outlines the capacity strengths and weaknesses at NCDC and NCAH identified through the One Health assessment of the priority zoonotic diseases. Capacities have been divided into the five categories related to detection; laboratory capacity; response; prevention and control measures; and communication and coordination.

Sector	Capacity	Strengths Identified	Weaknesses Identified
NCDC	Detection	Case definitions for endemic diseases Procedures for sample collection	Case definitions for less common infectious diseases Effective reporting and surveillance systems for both endemic and emerging diseases
	Laboratory Capacity	Confirmation testing at the national reference laboratory	Rapid diagnostic testing at all levels Confirmation testing at governorate-level laboratories Private and public laboratory coordination on reporting results
	Response	Outbreak declaration criteria Subnational-level trained investigation team deployable to local level for outbreak response	Formal sub-national program and personnel dedicated to endemic and emerging infectious diseases Formalized outbreak response strategy Local-level personnel and training opportunities to support outbreak investigation and response
	Prevention and Control Measures	Collaboration with international organizations (FAO, WHO, WOAH)	Formal policies and procedures for vector and reservoir control measures Public education/awareness initiatives
	Communication and Coordination	Local-governorate-subnational-national-level communication and coordination International communication emerging infectious diseases reportable under IHR Annex 2	Multisectoral coordination International communication of endemic diseases
NCAH	Detection	Case definitions for endemic diseases Procedures for sample collection	
	Laboratory Capacity	Confirmation testing at the national reference laboratory	Rapid diagnostic testing at all levels Confirmation testing at governorate-level laboratories
	Response	Outbreak declaration criteria Formalized outbreak response strategy Subnational-level trained investigation team deployable to local level for outbreak response	Local-level personnel and training opportunities to support outbreak investigation and response
	Prevention and Control Measures	Formal policies and procedures for vector and reservoir control measures Collaboration with international organizations (FAO, WHO, WOAH) Public education/awareness initiatives	Financing for livestock vaccinations campaigns and mandated culling of infected animals
	Communication and Coordination	Local-governorate-subnational-national-level communication and coordination Multisectoral coordination International communication of endemic and emerging infectious diseases	

## Discussion

### Impacts of COVID-19 in Libya

In our assessment, we learned of modifications and severe disruptions to Libya’s public health system and essential health services resulting from COVID-19 [32]. Similar to governments worldwide, attention and critical resources were redirected away from national priorities, including zoonotic diseases, to support the evolving epidemiological situation [33]. Prioritization of COVID-19 samples for laboratory testing resulted in a mounting backlog of samples in storage; reallocation of resources delayed clinical sentinel sites expansion for communities outside of Tripoli; and discontinuation of annual nationwide vaccination campaigns left the public at an increased risk for infection and community spread of disease. Lapses in routine preventive measures for known endemic or epidemic-prone diseases undermine health system strengthening achievements and leave nations vulnerable to a plethora of risks from inadequate surveillance, laboratory capacity, and immunization. Therefore, future prevention and response strategies should emphasize integration, building on existing capacities rather than redirection of critical resources essential to combatting known disease threats.

### Detection and surveillance

Timely and sensitive human and animal health surveillance and alert systems are pivotal to the stable and functional public health systems and response operations. For nations experiencing prolonged humanitarian emergencies and conflict, routine surveillance capacity can be severely challenged [34, 35]. Presently, brucellosis case reports often require local-level validation and confirmation by NCDC. This requirement for diagnostic confirmation at the central level places unnecessary delays on case confirmation and the initiation of outbreak response and control measures; this delay could introduce inaccuracies to national epidemiological monitoring as well as burden of disease estimates. Therefore, our short-term recommendations reinforce training and education on reporting guidelines for clinicians, laboratories and local health officials to support efficiency of the passive surveillance system [36, 37]. Efforts to bolster reporting compliance should acknowledge endemic and emerging zoonotic risks factors to local communities, particularly rural areas in Libya, due to increased exposure to livestock and wildlife and consumption of raw milk products [38, 39]. Inadequate reporting delays detection and response in these high-risk locations and increases the opportunity for larger-scale outbreaks due to unrecognized disease spread and human and animal travel and trade movement [40–42].

In Libya, it is assumed that RVF transmission to humans is rare due to a lack of existing data, both clinical and epidemiological, creating a negative feedback loop of delayed or no laboratory confirmation, clinical diagnosis or treatment. Additionally, NCAH lacks a dedicated surveillance system for RVF. Instead of addressing capacity gaps for brucellosis and RVF separately, there are opportunities to bolster and sustain the existing endemic surveillance system and leverage capacities to support RVF case identification in humans and animals [38]. More robust and consistent reporting of brucellosis from both sectors would strengthen baseline case incidence data, which could be crucial in detecting and identifying unusual disease events, including RVF [43]. Additionally, building new, separate systems for emerging diseases is a challenge for low-resource areas as there is limited funding and human resources to that would need to be redirected away from endemic diseases [38, 44]. Regardless of the surveillance approach, it is essential that the human and animal health sectors communicate and share case information to support effective detection and subsequent response. The limitations we identified in Libya’s current human and animal health surveillance systems further challenge effective and efficient detection of both brucellosis and emerging infectious diseases like RVF in the Eastern Mediterranean region (EMR).

### Laboratory capacity

While strides have been made to expanded molecular diagnostics in Libya, ELISA and PCR testing capacity remains concentrated at the NCDC and NCAH national reference laboratories in Tripoli, placing reliance on the central-level for detection and diagnosis of brucellosis and RVF. Decentralizing diagnostic capacity and creating regional laboratories capable of running ELISA and PCR would help alleviate pressure on the national reference laboratories, particularly during outbreaks, and could expedite sample processing and case confirmation. In order to develop regional-level laboratory capacity, however, adequate budget for consumables, equipment, and personnel training must be allocated by NCDC and NCAH. Expansion to the regional or governorate level should prioritize high-risk areas with previous or known transmission, such as rural areas where human-animal interactions are more likely to occur. Private laboratories’ extensive diagnostic capacity also supports rapid, local-level disease detection and diagnosis; however, the test results are not shared with NCDC or NCAH or support case identification and sentinel surveillance. Therefore, ministries should leverage existing frameworks for private sector laboratory engagement in order to initiate discussions and outline parameters for a formalized partnership to expand access to testing capacities in the event of public or veterinary health events and strengthen national surveillance for both brucellosis and RVF [10, 45].

### Response

After the declaration of an outbreak of brucellosis or possible RVF, NCDC and NCAH coordinate, review control strategies and initiate response plans in the affected communities. We also identified coordination with other external sectors, such as law enforcement and security entities; while these findings are outside of the scope of this study, this multisectoral coordination is imperative to effective local outbreak control and response implementation [16]. Purposeful multisectoral engagement between the human and veterinary health sectors demonstrates Libya’s investment in One Health approaches to zoonotic disease response operations; nevertheless, the lack engagement with EGA across all of the priority diseases is an evident gap to implementing a comprehensive and holistic One Health strategy.

The Monitoring and Evaluation Department at NCDC has a RRT and along with the Zoonotic Disease Control Department deploys from Tripoli to lead local investigations and educate the public on risk factors to prevent further infection and spread of brucellosis; capacity is available for RVF outbreak management response, as well. NCAH is in the processes of developing a RRT through its Zoonotic Disease Control Unit providing the perfect opportunity to liaise with NCDC’s RRT, leverage their experience to design an integrated One Health RRT for both endemic and emerging zoonotic threats. Neither sector in Libya has subnational or local level rapid response capacity, a requirement prescribed in WHO’s Strategic Framework for EIDs in the EMR (2020–2024) and the IHR (2005) [5, 46]. As Libya develops joint, multisectoral RRTs for zoonoses, it will be important to include the impact of animal husbandry practices, abattoirs, exposure to wildlife, climate change, cultural practices and socio-economic conditions that impact zoonotic risk from Tripoli, to Sabhā, to Benghazi in the development of training modules and response plans. Unfortunately international curriculum models and trainings for health emergency response operations are disease-specific, requiring countries to adapt trainings for new diseases of concern [47]. We recommend creating disease-agnostic trainings and guidelines for health emergencies and extending these educational opportunities to local public and veterinary health officials to further strengthen community outbreak response and control.

### Prevention and control measures

Risk communication is a vital component in the event of an outbreak or epidemic. NCAH conducts routine educational campaigns to increase community awareness of brucellosis through radio broadcasts, brochures, and leaflets, however NCDC lacks public awareness campaigns for brucellosis and RVF. While NCAH has developed numerous policies and procedures for brucellosis and RVF containment and control, there is a lack of adequate finances to fund immunizations and repay farmers for mandated culling of infected animals. Libya’s recurrent political and economic challenges have only further hindered implementation of sustainable prevention and control measures. From these findings, we recommended that NCDC and NCAH initiate joint risk communications for routine public awareness campaigns targeting high-risk populations, farmers, abattoirs, and veterinarians on transmission risks, symptoms and preventive measures for both brucellosis and RVF. These joint efforts should include targeted messaging using a systems approach: communications through several media platforms, (TV, radio, print, etc.), community partnerships, and engagement in activities led by local organizations embedded within the target community [41, 42]. Additionally, necessary funding needs to be properly budgeted and allocated to reinstate compensation to farmers for culled animals, as well and for immunizations campaigns. Research concludes that the combination of vaccination, culling of infected animals, environmental sanitation, and personal protection in humans are both cost-effective and significantly reduce zoonotic disease spread in both humans and animals [48].

## Conclusions

Libya’s One Health infrastructure has faced enormous challenges over the past decade, but retains strong capabilities particularly related to endemic diseases, which provide opportunities for system-wide strengthening and improved ability to address emerging threats. Operationally, the One Health assessment of priority zoonoses in Libya was successfully completed despite exceptional and often uncertain circumstances. The multi-country research team maintained remote correspondence throughout the duration of the project and persistently maintained flexibility in our timelines and expectations. Many of our Libyan stakeholders, as well as co-authors, faced overwhelming pressures and balanced multiple responsibilities due to the surge response to COVID-19. Nevertheless, we found that the virtual environment proved to be an effective means of communicating and collaborating. Regardless of the circumstances, there is a clear dedication from both NCDC and NCAH in Libya to develop sustainable health security capacity and work concertedly to address zoonotic disease threats.

It is imperative that future global health security frameworks acknowledge the potential seismic impacts to health systems and provisions in response to the emergence of a novel disease, which historically has proven to paradoxically redirect resources away from existing national priority diseases, including zoonoses. While there has been a surge in health system strengthening efforts following the emergence of COVID-19, frameworks and metrics often neglect the challenges and significant impacts of conflict and sociopolitical issues on access to resources and sustainability of implementation in comparison to stable, high-income country settings. The need for adaptable models and flexible assessment tools to meet the unique and uncertain challenges of conflict settings is imperative. In addition, with the emergence and re-emergence of zoonoses, the imperative push for more One Health integration and implementation should be adjustable, acknowledging the limitations of low-resource settings. It is imperative that critical lessons learned from COVID-19 response are recognized in preparation for future novel diseases.

## Supporting information

S1 Fig Systems Map Schematic for leishmaniasis from the community level (top) to the international level (bottom). The figure depicts a flow chart schematic of surveillance and laboratory mapping for leishmaniasis. Efforts in surveillance and response led by NCDC are represented in dark blue while those led by NCAH and EGA are in green and light blue, respectively. Abbreviations: NCDC = National Centre for Disease Control; NCAH = National Centre for Animal Health; EGA = Environment General Authority; MOH = Ministry of Health; MOA = Ministry of Agriculture; MOE = Ministry of Environment. Solid arrows represent sample/patient sharing. Arrows with dashes represent information sharing. Dark blue arrows indicate human cases, samples, and/or shared information whereas green and light blue arrows show animal-related information and wildlife-related information, respectively. Red arrows with dashes indicate current gaps.
(TIFF)

S2 Fig Systems Map Schematic for Highly Pathogenic Avian Influenza (HPAI) from the community level (top) to the international level (bottom). The figure depicts a flow chart schematic of surveillance and laboratory mapping for HPAI. Efforts in surveillance and response led by NCDC are represented in dark blue while those led by NCAH and EGA are in green and light blue, respectively. Abbreviations: NCDC = National Centre for Disease Control; NCAH = National Centre for Animal Health; EGA = Environment General Authority; MOH = Ministry of Health; MOA = Ministry of Agriculture; MOE = Ministry of Environment. Solid arrows represent sample/patient sharing. Arrows with dashes represent information sharing. Dark blue arrows indicate human cases, samples, and/or shared information whereas green and light blue arrows show animal-related information and wildlife-related information, respectively. Red arrows with dashes indicate current gaps.
(TIFF)

S3 Fig Systems Map Schematic for rabies case identification, diagnosis, and reporting in Libya from community level (top) to international level (bottom). The figure depicts a flow chart schematic of surveillance and laboratory mapping for rabies. Efforts in surveillance and response led by NCDC are represented in dark blue while those led by NCAH and EGA are in green and light blue, respectively. Abbreviations: NCDC = National Centre for Disease Control; NCAH = National Centre for Animal Health; EGA = Environment General Authority; MOH = Ministry of Health; MOA = Ministry of Agriculture; MOE = Ministry of Environment. Solid arrows represent sample/patient sharing. Arrows with dashes represent information sharing. Dark blue arrows indicate human cases, samples, and/or shared information whereas green and light blue arrows show animal-related information and wildlife-related information, respectively. Red arrows with dashes indicate current gaps.
(TIFF)
