# Supplementary figures and images for: Using One Health assessments to leverage endemic disease frameworks for emerging zoonotic disease threats in Libya
Source: PLOS Glob Public Health. 2023 Jul 26;3(7):e0002005. doi: 10.1371/journal.pgph.0002005 (PMC10370693; doi:10.1371/journal.pgph.0002005)

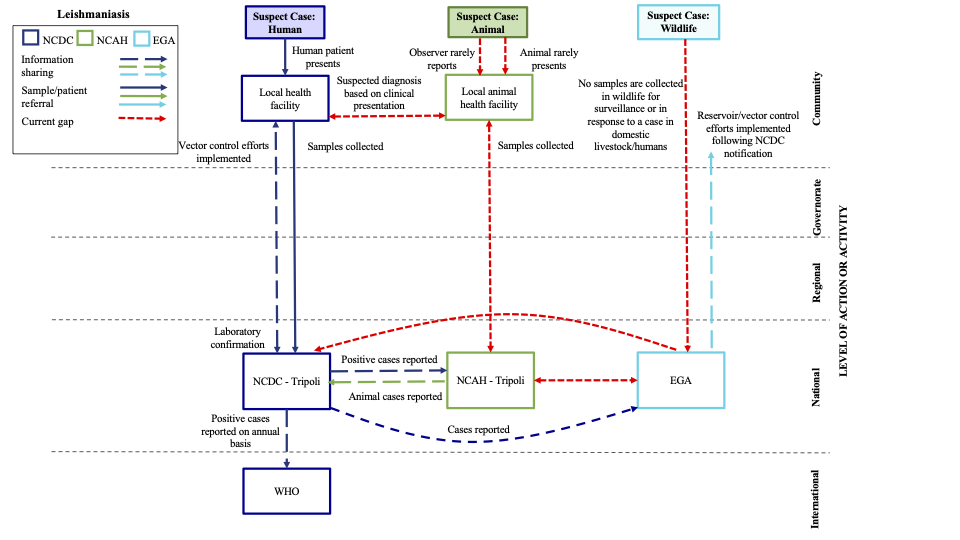

Supplement: S1 Fig — Systems Map Schematic for leishmaniasis from the community level (top) to the international level (bottom). The figure depicts a flow chart schematic of surveillance and laboratory mapping for leishmaniasis. Efforts in surveillance and response led by NCDC are represented in dark blue while those led by NCAH and EGA are in green and light blue, respectively. Abbreviations: NCDC = National Centre for Disease Control; NCAH = National Centre for Animal Health; EGA = Environment General Authority; MOH = Ministry of Health; MOA = Ministry of Agriculture; MOE = Ministry of Environment. Solid arrows represent sample/patient sharing. Arrows with dashes represent information sharing. Dark blue arrows indicate human cases, samples, and/or shared information whereas green and light blue arrows show animal-related information and wildlife-related information, respectively. Red arrows with dashes indicate current gaps. (TIFF) [file pgph.0002005.s001.tiff]

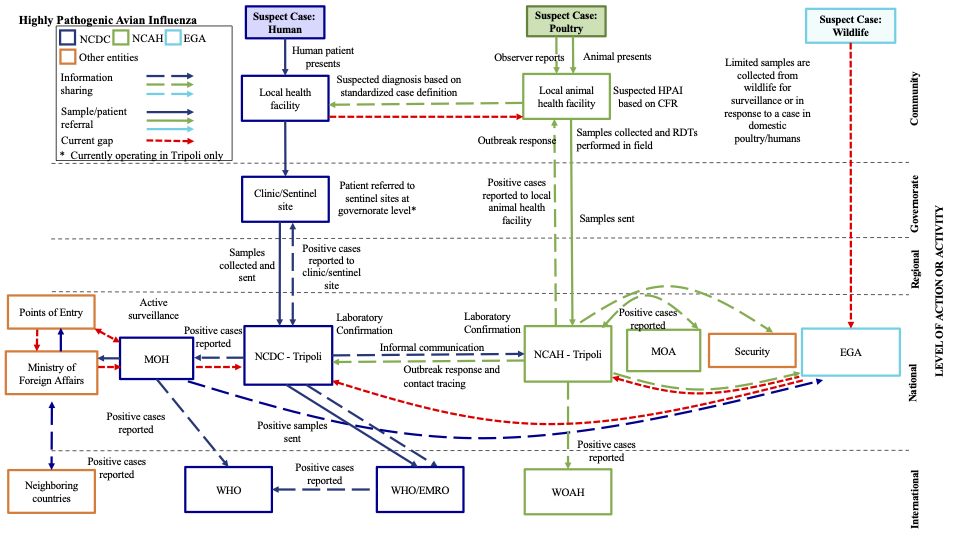

Supplement: S2 Fig — Systems Map Schematic for Highly Pathogenic Avian Influenza (HPAI) from the community level (top) to the international level (bottom). The figure depicts a flow chart schematic of surveillance and laboratory mapping for HPAI. Efforts in surveillance and response led by NCDC are represented in dark blue while those led by NCAH and EGA are in green and light blue, respectively. Abbreviations: NCDC = National Centre for Disease Control; NCAH = National Centre for Animal Health; EGA = Environment General Authority; MOH = Ministry of Health; MOA = Ministry of Agriculture; MOE = Ministry of Environment. Solid arrows represent sample/patient sharing. Arrows with dashes represent information sharing. Dark blue arrows indicate human cases, samples, and/or shared information whereas green and light blue arrows show animal-related information and wildlife-related information, respectively. Red arrows with dashes indicate current gaps. (TIFF) [file pgph.0002005.s002.tiff]

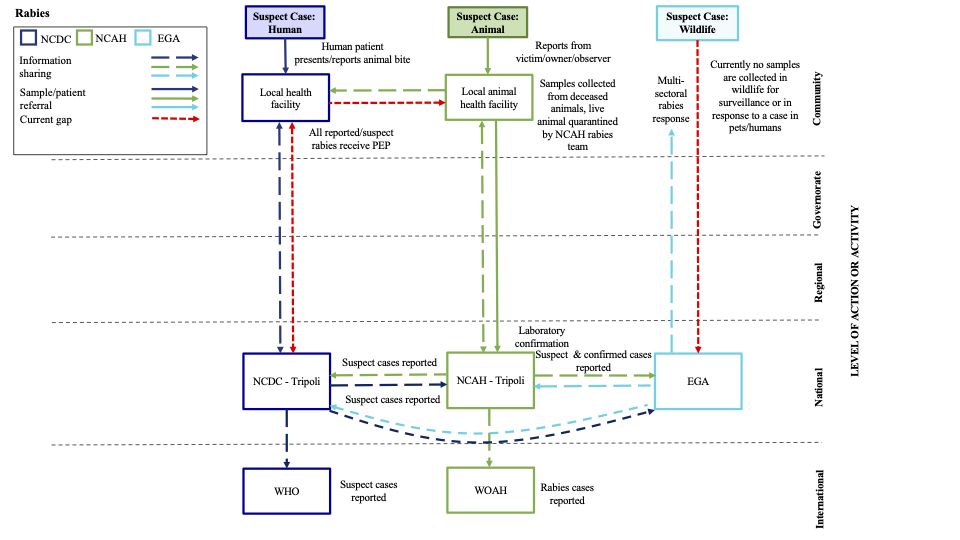

Supplement: S3 Fig — Systems Map Schematic for rabies case identification, diagnosis, and reporting in Libya from community level (top) to international level (bottom). The figure depicts a flow chart schematic of surveillance and laboratory mapping for rabies. Efforts in surveillance and response led by NCDC are represented in dark blue while those led by NCAH and EGA are in green and light blue, respectively. Abbreviations: NCDC = National Centre for Disease Control; NCAH = National Centre for Animal Health; EGA = Environment General Authority; MOH = Ministry of Health; MOA = Ministry of Agriculture; MOE = Ministry of Environment. Solid arrows represent sample/patient sharing. Arrows with dashes represent information sharing. Dark blue arrows indicate human cases, samples, and/or shared information whereas green and light blue arrows show animal-related information and wildlife-related information, respectively. Red arrows with dashes indicate current gaps. (TIFF) [file pgph.0002005.s003.tiff]
